# Association Between Baseline Blood Pressures, Heart Rates, and Vasovagal Syncope in Children and Adolescents

**DOI:** 10.7759/cureus.2119

**Published:** 2018-01-28

**Authors:** Himanshu Adlakha, Ruchi Gupta, Romana Hassan, Jeffrey H Kern

**Affiliations:** 1 Pediatrics, Pediatric Cardiology, Flushing Hospital Medical Center, Mount Sinai School of Medicine; 2 Pediatrics, Flushing Hospital Medical Center; 3 Pediatrics, Shadan Institute of Medical Sciences, India; 4 Pediatric Cardiology, Weil Cornell Medicine, New York Presbyterian Hospital, New York, Ny, Flushing Hospital Medical Centre

**Keywords:** vasovagal syncope, fainting, benign syncope, low baseline blood pressure, salt intake

## Abstract

Background: Vasovagal syncope is the most common cause of syncope in children and adults, accounting for 50-66% of unexplained syncope. There are no studies establishing the relationship between syncope, baseline heart rate, and blood pressure.

Objective: To identify a possible association between baseline blood pressure and heart rate with syncope.

Design/Methods: We conducted a questionnaire-based chart review study. A questionnaire was distributed to the guardian of children between eight and 18 years of age who attended the Pediatric Ambulatory Care Clinic at Flushing Hospital Medical Center. Based on the responses in the questionnaire, subjects were classified either as cases (positive for syncope) or controls (negative for syncope). Children and adolescents with neurological, cardiac, or any medical condition that can cause syncopal episodes were excluded from the study. Data collected from the questionnaire included age, gender, ethnicity, medical history, family history of syncope, and the amount of salt used in food. Anthropometric and vital signs for the current visit (height, weight, BMI, blood pressure, and heart rate) and vital signs from two previous visits were collected from electronic medical records. The data was analyzed using t-test and chi-square test with Microsoft Excel software (Microsoft Office Standard, v. 14, Microsoft; 2010); p<0.05 was considered significant.

Results: A total of 197 subjects were included in this study. There were 18 cases and 179 controls. Of the cases, (4/18) 22.2% were more likely to have a systolic blood pressure lower than the 10th percentile for their gender, age, and height as compared with controls (7/179) 3.9%, p = 0.003. The subjects with a history of syncope were more likely to add salt to their food (p = 0.004). There were no significant differences between cases and controls for age, gender, ethnicity between cases and controls for systolic blood pressure. No significant difference was observed between the heart rates of cases and controls.

Conclusions: Children and adolescents with syncope were more likely to have a systolic blood pressure lower than the 10th percentile, and there was no difference in the baseline heart rate. In addition, children with syncope were more likely to add salt to their food.

## Introduction

Syncope is defined as a sudden, brief loss of consciousness associated with loss of postural tone from which recovery is spontaneous [1]. It has been estimated that up to 15 percent of children experience a syncopal episode prior to the end of adolescence [2]. Although the etiology of syncopal events in children is most often benign, syncope occurring as the result of more serious diseases that are cardiac or neurologic have the potential for sudden death. The vast majority of the cases of syncope in the pediatric age group represent benign alterations in vascular tone [3]. This study will review the most common benign cause of syncope, i.e., vasovagal syncope.

Vasovagal syncope (also known as neurocardiogenic reflex, situational syncope, or common fainting) accounts for 50 percent or more of cases presenting to the emergency department. It is the most common cause of syncope, found in approximately 20 to 35 percent of cases, particularly in patients without any apparent neurologic or cardiac disease [4-9]. Typical clinical features would include a precipitating event and a prodrome. Precipitating events include standing as well as stress that could be physical or even emotional, although reflex precipitants such as swallowing, micturition, and hair grooming have also been reported [10-11]. Classical vasovagal syncope refers to syncope triggered by emotional or orthostatic stress such as venipuncture, either experiencing it or even witnessing the procedure; any painful or noxious stimuli; fear of bodily injury; prolonged standing; exertion or heat exposure. Prodrome would typically be described as lightheadedness causing dizziness, visual changes including decreased acuity, tunnel vision, or doubling of vision, nausea, pallor, and diaphoresis. The supine position helps in restoring adequate blood flow to the brain. However, immediate full recovery may sometimes be delayed as the patient may feel fatigued or depressed. This typical course of events is sometimes helpful sometimes to distinguish vasovagal syncope from syncope associated with arrhythmias which are usually [1] of abrupt onset and of short duration. Loss of consciousness may be prolonged with some other causes of syncope, such as seizures but rarely with vasovagal syncope. The diagnosis is clinical and based on specific history with known triggers, but not all patients would present with a classic history. The diagnosis can also be made by doing upright tilt table testing, during which the patient may pass out from bradycardia and/or hypotension or just by excluding all other causes. While the mechanism of syncope in patients with isolated tilt-positive syncope without any underlying cardiac or neurological condition has been extensively studied [12], possible associations with a baseline blood pressure level and heart rate have never been documented.

## Materials and methods

We conducted this cross-sectional chart review study by a questionnaire in either English or Spanish, from August 2014 to August 2015. Parents or guardians completed a questionnaire on behalf of their children aged between eight to 18 years. Of 197 completed questionnaires, 177 children were of Latin American descent, and remaining 20 children were either Caucasian or Asian. Children and adolescents with neurological, cardiac, or any medical condition that could cause syncope were excluded from the study. Data collected from the questionnaire includes age, gender, ethnicity, medical history, family history of syncope, triggering factor for syncope with prodromal symptoms of nausea, pallor, diaphoresis, lightheadedness and also the amount of salt used in the food. Based on the responses in the questionnaire, subjects were classified either as positive for syncope (cases) or negative for syncope (controls). Anthropometric measurements and vital signs from the current visit (height, weight, BMI, blood pressure, and heart rate) were collected from electronic medical records of the subjects. Vital signs from two previous clinic visits were also collected from the electronic medical records to document the consistency in the baseline blood pressure and heart rate readings. Systolic and diastolic percentiles for the blood pressures were then calculated from age and gender-specific nomograms. The data was analyzed using Microsoft Excel software (Microsoft Office Standard, v. 14, Microsoft; 2010), t-test, and chi-square; p<0.05 was considered significant.

## Results

**Figure 1:**
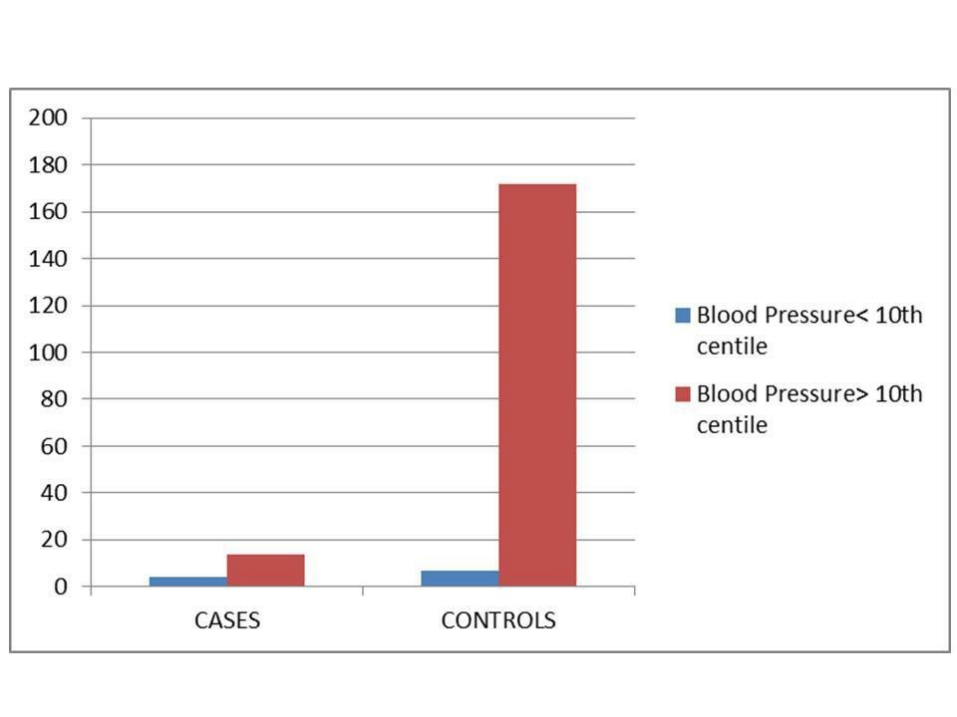
Comparison Between Baseline Blood Pressures: Cases vs Controls

A total of 197 subjects were included in this study. There were 18 cases (9.1% of the subjects) and 179 controls. There were no significant differences between cases and controls for systolic blood pressure, Four out of 18 cases, or 22.2%, were more likely to have a systolic blood pressure lower than the 10th percentile for their gender, age, and height as compared with controls (7/179) 3.9%, p = 0.003, as represented in Figure 1.

**Figure 2:**
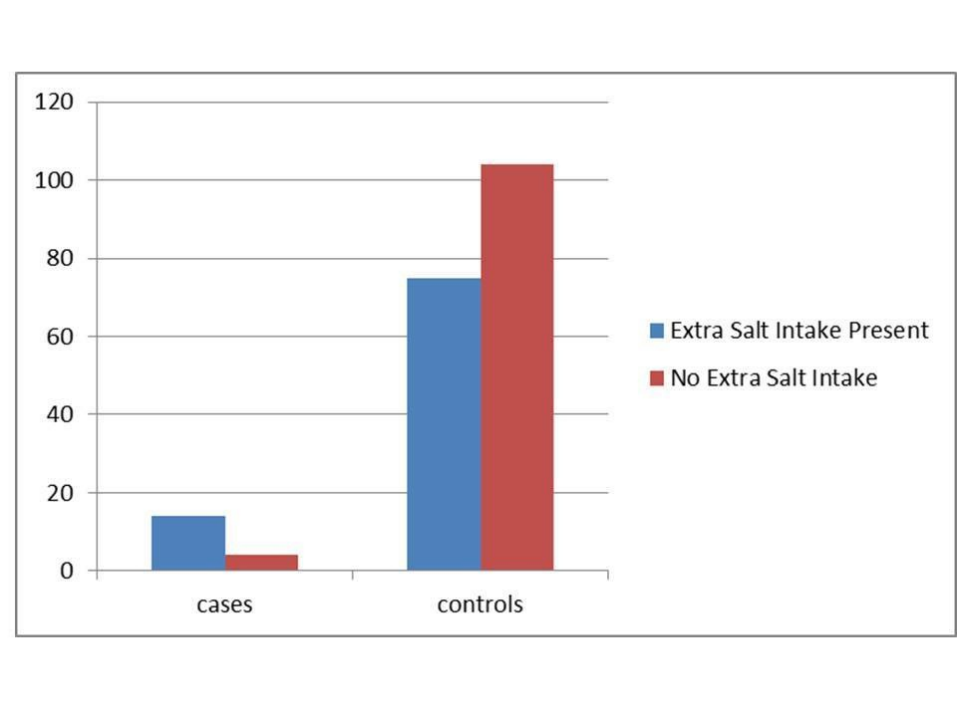
Comparison Between Salt Intake Levels: Cases vs Controls

Subjects with a history of syncope were more likely to add salt to their food (p = 0.004) as shown in Figure 2.

There were no significant differences between cases and control for age, gender, ethnicity, and observed heart rate.

## Discussion

Association between vasovagal syncope and lower baseline systolic blood pressures (less than 10th percentile for age and gender) provided insight into the nature, risks, and prognosis of the condition. The tendency to add more salt was found in those with a history of syncope, resulting in higher sodium levels in the blood which would cause more water retention and thus higher blood pressure. This could be described as an adaptive mechanism to involuntarily control the blood pressures and keep them in higher range so as to prevent any further episodes of the syncope. Various other preventive mechanisms have been well described and studied, such as assuming the supine position with leg raising at the onset of symptoms, avoiding trigger events, and medications that may induce hypotension. Counter-pressure maneuvers, such as arm tensing with clenching of fists, leg crossing, and leg pumping may help in aborting a syncopal event or at least delay it long enough for patients to assume the supine position. These maneuvers work by reducing lower-extremity venous pooling and improve cardiac output, thus preventing vasovagal syncope. The European Society of Cardiology guidelines (2009) noted that tilt training may be useful for the education of patients; its long-term benefits depend on compliance [13]. Additionally, it has been noted that symptoms resolve over time; a self-reported study on symptom burden in 418 patients diagnosed with vasovagal syncope indicated that 35 percent were symptom-free at the median five-year follow-up, regardless of presenting symptoms or treatment received [14].

## Conclusions

Appropriate management of vasovagal syncope can only come with a clear understanding of the complex pathophysiology of these episodes. Our study showed that children and adolescents with vasovagal syncope were more likely to have a systolic blood pressure lower than the 10th percentile when compared with controls, with no difference in the baseline heart rate. In addition, children and adolescents with syncope were more likely to add salt to their diet. These patients adapt over time to keep their blood pressures higher so as to prevent these syncopal episodes. Further studies and a larger sample size are needed.
